# A *BRCA1* deficient-like signature is enriched in breast cancer brain metastases and predicts DNA damage-induced poly (ADP-ribose) polymerase inhibitor sensitivity

**DOI:** 10.1186/bcr3625

**Published:** 2014-03-14

**Authors:** Ryan P McMullin, Ben S Wittner, Chuanwei Yang, Benjamin R Denton-Schneider, Daniel Hicks, Raj Singavarapu, Sharon Moulis, Jeongeun Lee, Mohammad R Akbari, Steven A Narod, Kenneth D Aldape, Patricia S Steeg, Sridhar Ramaswamy, Dennis C Sgroi

**Affiliations:** 1 Molecular Pathology Unit, Massachusetts General Hospital, 73 High Street, Charlestown, MA 02129, USA; 2 Center for Cancer Research, Massachusetts General Hospital, 73 High Street, Charlestown, MA 02129, USA; 3 Department of Pathology, Harvard Medical School, 25 Shattuck Street, Boston, MA 02115, USA; 4 Department of Medicine, Massachusetts General Hospital, 55 Fruit Street, Boston, MA 02114, USA; 5 Department of Neurosurgery, Familial Breast Cancer Research, Women’s College Hospital Research Institute, 790 Bay Street, Toronto, ON M5G 1N8, Canada; 6 MD Anderson Cancer Center, Texas Medical Center, 2450 Holcombe Boulevard, Houston, TX 77021, USA; 7 Women’s Cancers Section, Laboratory of Molecular Pharmacology, National Cancer Institute, 9609 Medical Center Drive, Bethesda, MD 20892, USA

## Abstract

**Introduction:**

There is an unmet clinical need for biomarkers to identify breast cancer patients at an increased risk of developing brain metastases. The objective is to identify gene signatures and biological pathways associated with human epidermal growth factor receptor 2-positive (HER2+) brain metastasis.

**Methods:**

We combined laser capture microdissection and gene expression microarrays to analyze malignant epithelium from HER2+ breast cancer brain metastases with that from HER2+ nonmetastatic primary tumors. Differential gene expression was performed including gene set enrichment analysis (GSEA) using publicly available breast cancer gene expression data sets.

**Results:**

In a cohort of HER2+ breast cancer brain metastases, we identified a gene expression signature that anti-correlates with overexpression of *BRCA1.* Sequence analysis of the HER2+ brain metastases revealed no pathogenic mutations of *BRCA1*, and therefore the aforementioned signature was designated *BRCA1 Deficient-Like* (*BD-L*). Evaluation of an independent cohort of breast cancer metastases demonstrated that *BD-L* values are significantly higher in brain metastases as compared to other metastatic sites. Although the *BD-L* signature is present in all subtypes of breast cancer, it is significantly higher in *BRCA1* mutant primary tumors as compared with sporadic breast tumors. Additionally, *BD-L* signature values are significantly higher in HER2-/ER- primary tumors as compared with HER2+/ER + and HER2-/ER + tumors. The *BD-L* signature correlates with breast cancer cell line pharmacologic response to a combination of poly (ADP-ribose) polymerase (PARP) inhibitor and temozolomide, and the signature outperformed four published gene signatures of *BRCA1/2* deficiency.

**Conclusions:**

A *BD-L* signature is enriched in HER2+ breast cancer brain metastases without pathogenic *BRCA1* mutations. Unexpectedly, elevated *BD-L* values are found in a subset of primary tumors across all breast cancer subtypes. Evaluation of pharmacological sensitivity in breast cancer cell lines representing all breast cancer subtypes suggests the *BD-L* signature may serve as a biomarker to identify sporadic breast cancer patients who might benefit from a therapeutic combination of PARP inhibitor and temozolomide and may be indicative of a dysfunctional *BRCA1*-associated pathway.

## Introduction

Central nervous system metastases are diagnosed in approximately 10% to 16% of women with advanced breast cancer [1,2]. The total incidence of brain metastases is potentially higher than currently reported statistics, as most brain metastases are diagnosed in response to clinical symptoms rather than by an initial detection. Several risk factors have been associated with the development of brain lesions in patients with metastatic breast cancer (MBC), including a younger age [3], having more than two metastatic sites at diagnosis [3], negative estrogen receptor (ER) status [1,4,5], human epidermal growth factor receptor 2-positive (HER2+) disease [1,4], and *BRCA1/2* mutation [6-8]. Survival for breast cancer patients with brain metastases is poor, with a one-year survival probability of approximately 20% [2]. These statistics highlight the crucial need to develop biomarkers for the prediction of brain metastasis risk and to identify the underlying biological pathways that promote brain metastasis for the development of potential targeted therapeutics.

Patients with HER2+ MBC tumors are two to four times more likely to develop brain metastases than patients with HER2-negative disease [1,4]. While systemic trastuzumab has proven efficacious for treating aggressive HER2+ breast cancer, its use has been associated with the central nervous system as the first site of relapse [9]. Thus, there is an urgent clinical need for biomarkers to identify patients at higher risk of developing brain metastases, as well as to identify alternative therapeutic approaches. In this study, we aim to identify gene signatures associated with HER2+ brain metastases for potential biomarker development as well as to provide insight into the underlying associated biological pathways.

## Materials and methods

### Patients and clinical samples

Patient and primary tumor characteristics are presented in Additional file 1. The HER2 status was assessed by HER2 immunohistochemistry (IHC) and/or gene amplification, and tumor grading was determined as described previously [10]. The breast cancer brain metastatic specimens consisted of fresh frozen biopsies obtained from the MD Anderson Cancer Center between 1998 and 2001; in all 19 cases the brain was the first site of relapse. As patient-matched primary breast tumor specimens were not available for these brain metastatic samples, we obtained HER2+ primary breast cancer specimens from Massachusetts General Hospital; these samples were obtained from patients with either no relapse or relapse to sites other than the central nervous system and consisted of fresh frozen biopsies obtained between 1998 and 2006. These breast cancer brain metastatic specimens and breast tumors were matched for patient age upon primary tumor detection and the ER status of the primary tumor. Patient consent was obtained for study participation and the study was approved by the human research committees of the MD Anderson Cancer Center and the Massachusetts General Hospital in accordance with the National Institutes of Health human research study guidelines.

### Laser capture microdissection, RNA extraction, and microarray hybridization

RNA was isolated from a highly enriched population of 4,000 to 5,000 malignant epithelial cells procured by laser capture microdissection and was hybridized to Affymetrix X3P GeneChips (Affymetrix, Santa Clara, CA, USA) as previously described [11]. The data was deposited in the National Center for Biotechnology Information (NCBI) Gene Expression Omnibus (GEO) [12] and are accessible through GEO Series accession number GSE43837 [13].

### Gene set enrichment analysis

Computation of gene expression was done using the MAS5 algorithm as implemented in the call.expers function in version 2.14.05 of the simpleaffy package of Bioconductor [14]. Gene set enrichment analysis (GSEA) analysis was performed using version 2.0 of GSEA run on all the gene sets in version 2.5 of the Molecular Signatures Database (MSigDB) [15].

### Calculation of *BRCA1 Deficient-Like* metagene value

All the genes in the BRCA1_OVEREXP_DN gene set, which was experimentally derived as described [16], in version 2.5 of the MSigDB [17] were mapped as described below to microarray identifiers. The gene expression values for all those identifiers were then averaged to form the *BRCA1 Deficient-Like* (*BD-L*) metagene. Specific probes measured are indicated in Additional file 1 for each figure.

### Mapping gene symbols to microarray identifiers

Gene symbols were mapped to Entrez GeneIDs using the 2 February 2008 version of the gene information file from *ftp.ncbi.nlm.nih.gov/gene/DATA*. First the ‘Symbol’ column was searched and, if that failed, the ‘Synonyms’ column was searched. To map an Entrez GeneID to Affymetrix HG-U133A probe set identifiers, version na24 of the annotation file from the Affymetrix website was used. The ‘Entrez Gene’ column of that annotation file was augmented by trying to fill empty entries by using the corresponding entries in the ‘UniGene ID’ and ‘Representative Public ID’ columns to search the file Hs.data from build 209 of Unigene and the 2 February 2008 version of the gene2accession file from *ftp.ncbi.nlm.nih.gov/gene/DATA*. An Entrez GeneID was then mapped to every probe set identifier that had the Entrez GeneID in the augmented ‘Entrez Gene’ column. To map Entrez GeneIDs to Rosetta spot IDs, we used [18] (downloaded 2 February 2008), the file Hs.data from build 209 of Unigene and the 2 February 2008 version of the gene2accession file from *ftp.ncbi.nlm.nih.gov/gene/DATA*.

### Sequencing of genomic *BRCA1*

All 22 coding exons of the *BRCA1* (NM_007294.3) gene were amplified and sequenced in 33 fragments using tumor DNA as previously demonstrated [19]. Primers were designed using Primer 3 software [20] to cover at least 20 base pairs at each 5’ and 3’ side of the exons. The amplified DNA fragments were sequenced by using the BigDye Terminator Cycle Sequencing kit on an ABI 3500xl DNA Analyzer (Applied Biosystems, Foster City, CA, USA). Sequencing chromatograms generated by the analyzer were examined for variant detection using Mutation Surveyor software (SoftGenetics LLC., State College, PA, USA).

### Statistical methods for correlative analyses

**Figure 1:**
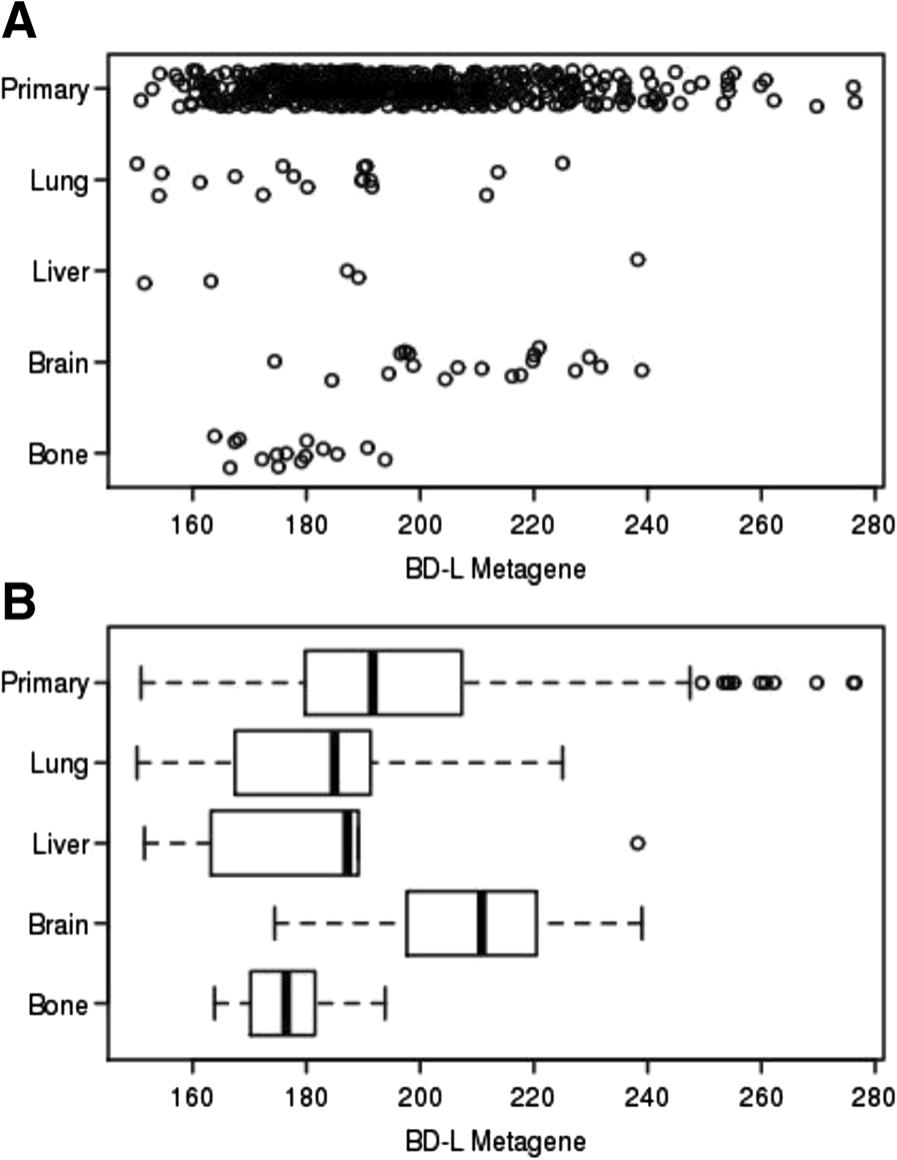
***BD-L *metagene values in primary and metastatic specimens.** Values in primary breast cancer and metastatic breast cancer samples as calculated from a previously described cohort detailed in Zhang *et al*. [29]. **(A)** Dot plot of individual metagene values. **(B)** Box plot of metagene values. Statistically significant differences in *BD-L* metagene value were observed for the brain metastases when compared to primary tumors (*P* value = 0.0043), bone metastases (*P* value = 4 × 10^-6^), and lung metastases (*P* value = 0.001). *BD-L, BRCA1 Deficient-Like.*

**Figure 2:**
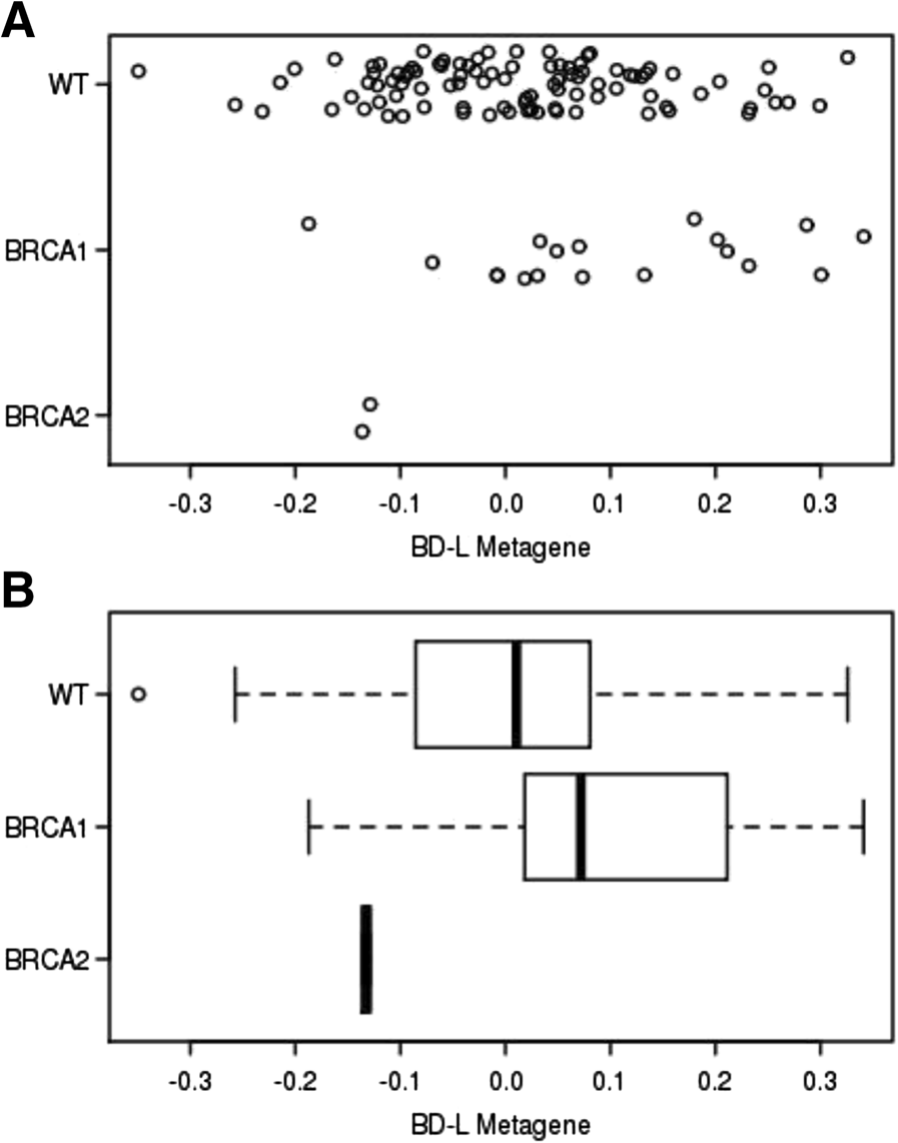
***BD-L *metagene values in sporadic and familial BRCA1 and BRCA2 primary tumors.***BRCA1* metagene values in primary sporadic breast cancer and BRCA1 and BRCA2 primary breast cancer samples as calculated from a previously described cohort detailed in van’t Veer *et al*. [30]. **(A)** Dot plot of individual metagene values. **(B)** Box plot of metagene values. A statistically significant difference in *BD-L* metagene value was observed for sporadic primary breast tumors when compared to mutant *BRCA1* carrier primary breast tumors (*P* value = 0.033). *BD-L, BRCA1 Deficient-Like.*

**Figure 3:**
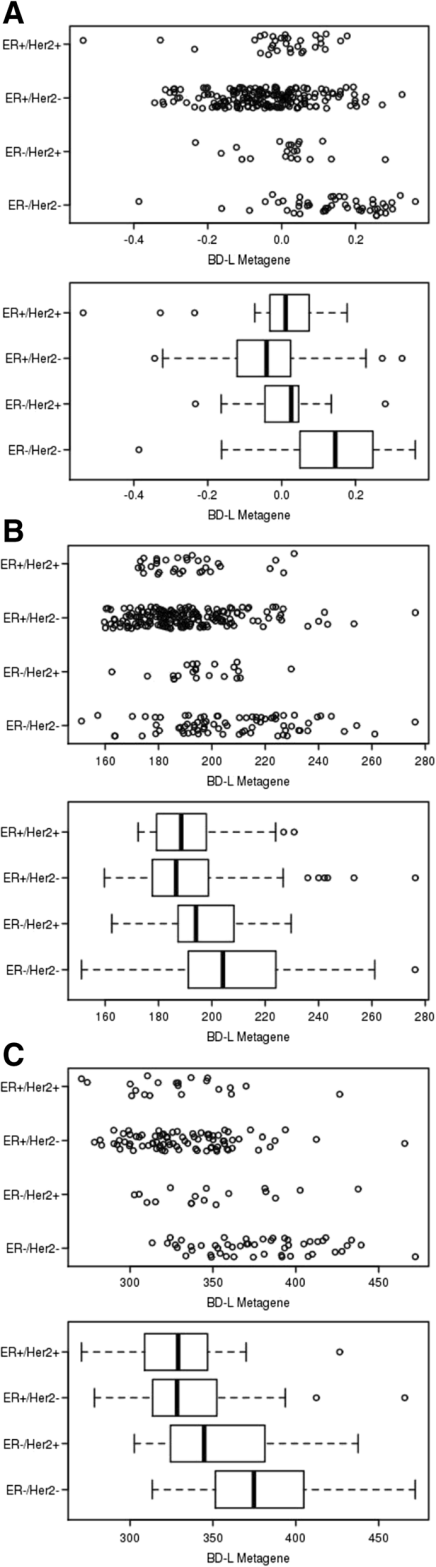
**Distribution of *BD-L *value by ER and HER2 status.** Dot plot and box plot distribution of *BD-L* metagene values by ER and HER2 status and correlation with clinical outcome in the **(A)** NKI295, **(B)** EMC286/MSK82, and **(C)** EMC192 cohorts. Statistically significant differences in *BD-L* metagene values were observed for ER-/HER- primary tumors when compared to ER+/HER2+ (*P* value = **(A)** 4.5 × 10^-6^, **(B)** 0.0025, **(C)** 2.8 × 10^-5^) and ER+/HER2- (*P* value = **(A)** 1.1 × 10^-13^, **(B)** 4 × 10^-8^, **(C)** 8.7 × 10^-10^) subgroups. *BD-L, BRCA1 Deficient-Like*; ER, estrogen receptor; HER2, human epidermal growth factor receptor 2*.*

The *P* values quoted for Figures 1, 2, and 3A were obtained by applying the Wilcoxon test to all pairs within the figure and correcting the resulting *P* values for multiple hypothesis testing using the Holm method [16].

### Cell culture and pharmacologic inhibition assay

All cell lines were obtained and maintained as previously described [21]. Independent pharmacologic inhibition assays were conducted in triplicate for each cell line. Cells were seeded at 20,000 per well in a 24-well plate. After 24 hours, cells were treated in triplicate with indicated concentrations of: DMSO, temozolomide (T2577, Sigma-Aldrich, St Louis, MO, USA), or AZD-2281 (S1060, Selleck Chemicals, Houston, TX, USA). After five days of incubation, cells were fixed in 4% formaldehyde and stained with 1% Crystal Violet (C0775, Sigma-Aldrich) for 10 minutes at room temperature. Cells were then washed to remove unincorporated dye and plates were inverted to dry overnight. Incorporated dye was extracted with 10% acetic acid and OD595 measurements were obtained within a linear range. Treated cells were normalized to the vehicle-treated control to obtain mean percentage viability.

## Results and discussion

To identify gene expression patterns that differentiate HER2+ breast cancer brain metastases from HER2+ primary tumors that did not metastasize to the brain, we performed a comparative gene expression analysis between 19 brain metastasis specimens from breast cancer patients with the brain as a first-site metastasis and 19 non-patient-matched primary breast tumor specimens from patients who either did not experience a relapse (with minimum follow-up time of >6 years) or did not have a recurrence in the central nervous system. None of the patients with a brain metastasis received herceptin; four of the patients without a relapse received neoadjuvant or adjuvant herceptin. These specimens were matched based upon the age of patient at initial detection, and the HER2 and ER status of the primary tumors (Additional file 1). Although patient-matched primary tumors were not available for comparative gene expression analysis, we hypothesized that the direct comparison of brain metastases to non-patient-matched primary tumors would provide insight into the key molecular pathways underlying outgrowth in the brain microenvironment.

To compare gene expression, RNA derived from microdissected tissue was hybridized to Affymetrix X3P GeneChips and the resulting data was subjected to bioinformatic analyses. Standard MAS5 pre-processing of the data with a *t*-test comparison and a false discovery rate set at 0.25 failed to identify individually differentially expressed genes between the brain metastatic specimens and the non-patient-matched primary breast cancer specimens.

As no significant differential expression for individual genes was discovered, a GSEA using version 2.5 the Broad Institute MSigDB was conducted to determine if there were modulations of gene sets that comprise annotated biochemical pathways [22]. The analysis yielded 22 enriched gene sets with a false discovery rate of q value <0.25 (Additional file 1). The top gene set identified was *BRCA1_OVEREXP_DN*, which is comprised of probe sets that were downregulated by two- to four-fold after inducible expression of BRCA1 in the BRCA1-low, ER + EcR-293 human embryonal kidney epithelial cell line [23]. A significant correlation of the HER2+ breast cancer brain metastases with a BRCA1-related signature was unexpected as several studies have reported a low frequency of HER2 expression in tumors of *BRCA1* mutation carriers [24-26], and thus may reflect more upon the underlying biology of metastatic outgrowth in the brain rather than an aspect of HER2 signaling. Furthermore, sequencing analysis for 17 of the 19 HER2+ brain metastatic specimens for which sufficient residual tumor remained identified no previously known pathogenic or novel potentially pathogenic variants (Frameshift insertion/deletion, nonsense or essential splicing site variants) as classified by International Agency for Research on Cancer (IARC) recommendations [27]. As our identified signature consisted of genes that were downregulated when BRCA1 was overexpressed, we hypothesized that the converse upregulation of these genes may indicate an underlying deficiency in the BRCA1 functional pathway, either directly through BRCA1 or indirectly through a cooperating factor. Since the *BRCA1_OVEREXP_DN* signature was enriched in HER2+ breast cancer brain metastases that did not have known or potentially novel *BRCA1* pathogenic variants, we designated the *BRCA1_OVEREXP_DN* signature as the ‘*BRCA1 Deficient-Like*’ (*BD-L*) metagene. *BD-L* metagene values were calculated for each specimen, and significant association of the metagene with the brain metastatic samples was confirmed (Additional file 2, left panel; *P* value = 0.0082). Additionally, a significant difference in BRCA1 expression on the microarray between the primary tumors and brain metastases was not observed for two probe sets (Additional file 2, middle and right panel). Because several of the brain metastasis samples were exhausted during the previous analyses, a direct characterization of BRCA1 mRNA and protein expression was prohibited. However, a significant correlation between the two BRCA1 probe sets on the Affymetrix X3P GeneChip and the *BD-L* metagene was not observed for the entire cohort, suggesting that metagene value may not be merely tracking with BRCA1 mRNA expression (Additional file 3). While BRCA1 protein expression by IHC could not be examined, a previous study has suggested significant concordance between BRCA1 mRNA and protein expression in clinical specimens [28].

Although all patients in this cohort were confirmed to have clinical 3+ HER2+ breast cancer by IHC and/or fluorescent *in situ* hybridization (FISH), the possibility existed that they were misclassified. To confirm overexpression of HER2 across the cohort, the expression levels for all genes on the microarray were plotted on a histogram and indicated genes were denoted for each patient by a red line in Additional file 4. The expression of HER2 showed a clear enrichment compared to PSA, which is not reported to be highly expressed in breast cancer, and PRY, DAZ4, and CDY1, which are all located on chromosome Y and thus are not detected at high levels in female breast cancer samples. Thus, the consistently high level of HER2 expression across the cohort supports the clinical HER2+ diagnosis.

To validate our original observation that the *BD-L* metagene is enriched in breast cancer-derived brain metastases, gene expression data from an independent cohort consisting of 615 primary breast cancer specimens as well as breast cancer metastasis specimens from brain (n = 19), lung (n = 18), liver (n = 5), and bone (n = 15) was assessed for correlation with *BD-L*[29]. As demonstrated in Figure 1, a higher mean *BD-L* metagene value was observed in metastases to the brain as compared to primary tumors (*P* value = 0.0043), bone metastases (*P* value = 4 × 10^-6^), and lung metastases (*P* value = 0.001), but not when compared to liver (*P* value = 0.38). A limitation in using this data set is the restricted number of metastatic samples in each group and the lack of annotation of ER- and HER2-receptor status for the metastatic data points. Given this limitation, the significant association observed may support the enrichment of the *BD-L* signature as being a feature of brain metastases irrespective of receptor subtype.

Having confirmed an enrichment of *BD-L* metagene value in brain metastases compared to primary tumors, we then hypothesized that a metagene of *BRCA1* deficiency would also demonstrate increased values in primary tumor specimen derived from mutant *BRCA1* carriers compared to noncarriers. When a publicly available gene expression data set was interrogated [30], a significantly higher mean *BD-L* value was found in mutant *BRCA1* tumors (*P* value = 0.033) when compared to sporadic tumors (Figure 2). While the *BD-L* value in primary tumors between sporadic breast cancer patients and *BRCA2* mutation carriers was not significant, there is little power in the analysis given the small sample size (n = 2). The *BD-L* values for sporadic primary tumors included a subset with elevated metagene values comparable to those of *BRCA1* mutation carriers, which may be indicative of a subpopulation of sporadic tumors with characteristics similar to *BRCA1* mutated tumors. The correlation of the *BD-L* signature with both brain metastases and *BRCA1* mutation is consistent with the published literature as *BRCA1* mutation carriers are reported to have an increased prevalence of breast cancer brain relapse as compared to noncarriers [8,31].

To investigate the correlation of the *BD-L* metagene with important molecular markers of sporadic breast cancer subtypes, we next evaluated the distribution of *BD-L* value by HER2 and ER status in the NKI295 [32], EMC286 [33]/MSK82 [34], and EMC192 [35] cohorts of sporadic primary tumors (Figure 3A-C). As *BRCA1* mutants represent a subpopulation within the triple negative breast cancer, an expected significantly higher *BD-L* metagene mean value was observed in ER-/HER- primary tumors when compared to ER+/HER2+ subgroups, with *P* values = 4.5 × 10^-6^ (NKI295), 0.0025 (EMC286/MSK82), and 2.8 × 10^-5^ (EMC192). Additionally, *BD-L* mean value was significantly higher in ER-/HER- tumors when compared and ER+/HER2- tumors, with *P* values = 1.1 × 10^-13^ (NKI295), 4 × 10^-8^ (EMC286/MSK82), and 8.7 × 10^-10^ (EMC192). Although not consistently significant across the cohorts, a trend is observed when comparing ER-/HER- tumors to ER-/HER2+ tumors, with *P* values = 0.0023 (NKI295), 0.097 (EMC286/MSK82), and 0.05 (EMC192). Despite the significant correlation with a negative ER and/or HER2 receptor expression, it was notable that a small subpopulation of tumors with high *BD-L* values was present within the ER + and HER2+ subtypes (Figure 3A-C dot plots), suggesting that the *BD-L* phenotype may extend beyond primary tumors of *BRCA1* mutation carriers and the sporadic ER-/HER2- subtype. This is especially intriguing for primary ER + tumors because the brain is not a prevalent metastatic site for the ER + subtype [36]. Motivated by the possibility that the *BD-L* signature may extend across current breast cancer classifications of receptor expression or mutational status, we next sought to apply the *BD-L* signature to breast cancer cell lines independent of receptor and mutational status with an aim to identify a phenotype of pharmacological sensitivity.

We hypothesize that the *BD-L* metagene may identify breast cancers that fall within a spectrum of dysfunction for a BRCA1 functional complex or regulated pathway, either directly through BRCA1 or indirectly through a cooperating factor. Having demonstrated that *BD-L* was enriched in *BRCA1* mutation carriers, we hypothesized that breast cancer cell lines with elevated *BD-L* values may exhibit increased sensitivity to therapeutic agents that target a dysfunctional *BRCA1*-associated pathway. Poly (ADP-ribose) polymerase (PARP) inhibitors represent an exciting class of drugs that have demonstrated promise in clinical *BRCA1/2-*related cancers as single agents [37,38] and in preclinical studies as single agents and in combination with certain classes of DNA-damaging agents [39,40]. Additionally, preclinical testing has revealed that disruption of proteins that cooperate either directly or indirectly with BRCA1/2 proteins can increase PARP inhibitor sensitivity [41-43]. Because we hypothesize the *BD-L* metagene may correlate with a spectrum of dysfunction, we chose to induce DNA damage to enhance the effectiveness of the PARP inhibitor. Therefore, we tested a panel of breast cancer cell lines for sensitivity to a combination treatment with the PARP inhibitor olaparib (AZD-2281), an oral PARP inhibitor in clinical use that has shown evidence of crossing the blood/brain barrier [44], and the DNA alkylating/methylating agent temozolomide, a clinically utilized chemotherapeutic that can cross the blood/brain barrier and has demonstrated increased efficacy in combination with a PARP inhibitor [45-48]. Using a publicly available gene expression set, we determined *BD-L* metagene values for 51 well-defined human breast cancer cell lines as described in Neve *et al*. (Additional file 1) [21]. We rank-ordered the lines by increasing metagene value, and selected 12 cell lines predicted to be among either the most resistant or most sensitive to pharmacologic inhibition (Table 1). This panel included the *BRCA1*-deficient HCC1937 cell line, which the *BD-L* metagene predicts to exhibit low sensitivity. While this may appear paradoxical, clinical trials have demonstrated that not all *BRCA1* mutation carriers are responsive to PARP inhibitors [37,38]. Additionally, Figure 2A demonstrated that although the *BD-L* metagene is enriched in *BRCA1-*mutation carriers compared to noncarriers, a subset of *BRCA1* mutation carriers have low metagene values. Because we hypothesize the *BD-L* metagene provides a measure of a BRCA1-associated pathway function rather than a *BRCA1* gene mutation or the expression status, the metagene would also account for potential compensatory mechanisms.

**Table 1:** ***BRCA1 Deficient-Like *** **( *BD-L *) distribution in a breast cancer cell line panel**

**Cell line**	**ER**	**PR**	**HER2**	**BRCA1**	***BD-L *** **metagene value**
HCC1143	-	-	-	NA	207.2555
MDAMB415	+	-	-	wt/-	208.6937
HCC1937	-	-	-	m/-	226.181
BT20	-	-	-	wt/-	227.6686
MDAMB468	-	-	-	wt/-	260.2652
BT474	+	+	+	wt/-	261.2111
HCC1428	+	+	-	NA	265.375
MDAMB134VI	+	-	-	wt/wt	267.9736
SKBR3	-	-	+	wt/-	277.6819
MCF10A	-	-	-	NA	285.3522
HCC1954	-	-	+	NA	300.0448
HCC1500	-	-	-	NA	300.3202

Breast cancer cell lines selected for pharmacological inhibition evaluation. *BD-L* metagene values for the cell lines, as calculated from the Neve *et al*. [21] data set, are listed by increasing value. Estrogen receptor (ER), progesterone receptor (PR), and human epidermal growth factor receptor 2 (HER2) protein expression status are indicated. If known, the *BRCA1* mutational status is noted (NA: not available).

**Figure 4:**
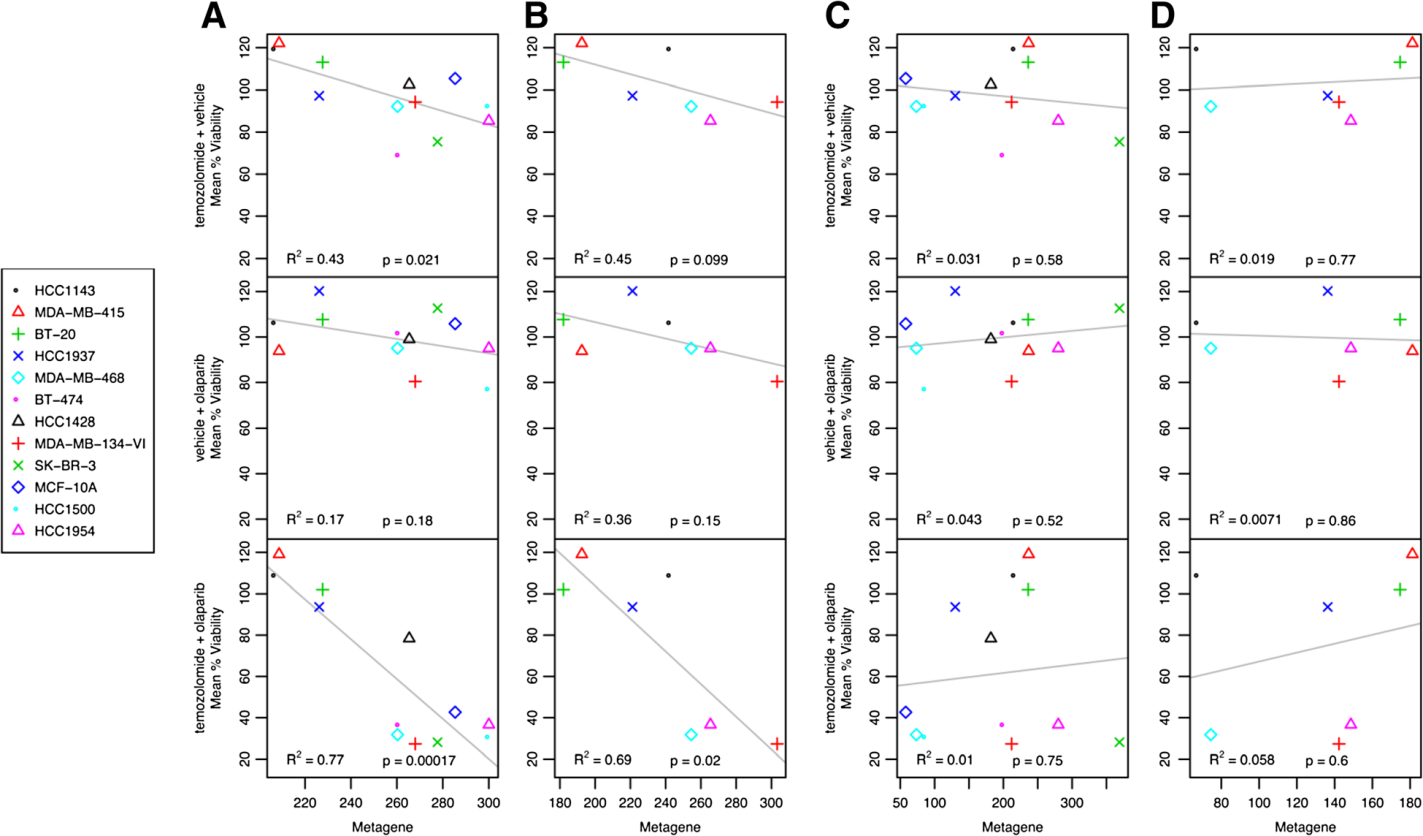
**Association of *BD-L *value with response to a combination of temozolomide and olaparib.** Linear regression analyses of single and combination treatment with 40 nM olaparib and 100 uM temozolomide using: **(A,B)***BD-L* metagene values calculated using datasets from **(A)** Neve *et al*. [21] and **(B)** Garnett *et al*. [50]; **(C,D)***BRCA1* breast cancer signature as described in van’t Veer *et al*. [30] calculated using data sets from **(C)** Neve *et al*. and **(D)** Garnett *et al. BD-L, BRCA1 Deficient-Like.*

Based upon known mechanisms of temozolomide-specific sensitivity and extensive *in vitro* pharmacological studies in cell lines [49], 100 uM was determined to be a physiologically relevant dose that does not demonstrate significant reduction in viability across the breast cancer cell line panel (Figure 4A, top panel). Single treatment and combined treatment with temozolomide using increasing sub-physiological doses of olaparib identified significant inhibition upon combination treatment. (Figure 4A, Additional file 5). As originally hypothesized, there is a highly significant correlation (R^2^ = 0.77; *P* value 0.00017) of the *BD-L* metagene with pharmacological response of cell lines to the combined administration of olaparib and temozolomide (Figure 4A, lower panel). It is interesting to note that the metagene was able to correctly predict the response of the *BRCA1*-deficient HCC1937 cell line, suggesting the *BD-L* metagene may be a better indicator of pharmacological response than *BRCA1* gene status. To further support the correlation with sensitivity, *BD-L* metagene values were calculated for seven of the tested cell lines from an independent gene expression data set described in Garnett *et al*. [50] and was plotted using our experimentally derived pharmacological response data. While single administration of either temozolomide or olaparib alone (Figure 4B, top and middle panel) did not demonstrate a significant reduction in viability, a significant correlation (R^2^ = 0.69; *P* value 0.02) is observed upon dual administration and supports our original observation (Figure 4B, lower panel). Thus, using two independent gene expression data sets of cell lines derived from different microarray platforms, the *BD-L* metagene demonstrated a strong correlation with our experimentally derived DNA damage-induced PARP inhibitor sensitivity.

To determine the robustness of *BD-L* metagene in predicting sensitivity, we evaluated the performance of five published signatures [30,51-53] of *BRCA1/2* deficiency and/or function in predicting our observed pharmacologic responses of the breast cancer cell line panel using the gene expression data from Neve *et al*. [21] (Figure 4C, Additional file 6A, C, E, G) and Garnett *et al*. [50] (Figure 4D, Additional file 6B, D, F, H). In contrast to the *BD-L* metagene (Figure 4A and B, bottom panels), all five *BRCA1/2-*related signatures failed to correlate with pharmacologic response (Figures 4C, D, Additional file 6A-H). The difference in predictive power is potentially due to the approach taken in discovering these signatures. The *BD-L* metagene was derived from changes in gene expression due to a modest overexpression of *BRCA1* within a single cell line. This unbiased approach goes beyond indicating the *BRCA1* mutational status or the acute response to a stimulus to provide a measure of BRCA1 pathway function that can include the contribution of BRCA1 and its interacting components. Alternatively, the genes that comprise the *BD-L* metagene may comprise functional networks that contribute to the observed PARP inhibitor sensitivity. Mapping of the 112 BD-L genes to functional networks using Ingenuity Pathway Analysis (IPA) identified a predominant network association with biological functions of proliferation, cell cycle control, and apoptosis (Additional file 1). While these functions have not previously been associated with response to PARP inhibitors, the potential for specific aspects of these functions for influencing sensitivity provide possible avenues for future investigation. In conclusion, the *BD-L* metagene may provide a measure of BRCA1 pathway function as opposed to indicating *BRCA1* mutational status, direct expression levels, or response to an acute stimulus.

## Conclusions

In summary, we identified a *BRCA1 Deficient-Like* metagene that is enriched in HER2+ brain metastases when compared with HER2+ primary tumors, and in an independent data set confirmed the enrichment of the metagene in brain metastases as compared to bone metastases, lung metastases, and primary breast tumors. Furthermore, we demonstrated that high *BD-L* metagene value is enriched in, but not limited to, primary tumors of *BRCA1* mutation carriers and sporadic ER-/HER2- patients. When the *BD-L* signature is calculated for a breast cancer cell line panel using gene expression from two independent data sets, the *BD-L* metagene correlates with pharmacologic response to a combination treatment of olaparib and a temozolomide. Lastly, we demonstrated that the *BD-L* metagene outperforms extant classifiers of *BRCA1/2* status in predicting pharmacological response to the drug combination in the breast cancer cell panel.

Since the clinical administration of PARP inhibitors is still in its infancy, there is a crucial need to both identify patients who will gain benefit from this class of drugs and to develop biomarkers that predict clinical response. Currently, *BRCA1/2* status is the prevailing indicator of potential PARP inhibitor sensitivity, although not all *BRCA1/2* breast cancers respond and there is preclinical evidence to suggest that PARP inhibitors may hold benefit in cancer populations beyond *BRCA1/2* mutation carriers [54]. Herein, we provide evidence that the *BD-L* metagene may be enriched in clinically detectable breast cancer brain metastases and the metagene may implicate sporadic breast cancers across the conventional receptor and mutational status classifications that may benefit from a PARP inhibitor-based therapy while also identifying triple negative and *BRCA1*-mutant cancers that may prove refractory to treatment.

## Abbreviations

BD-L: ‘*BRCA1 Deficient-Like*’; ER: estrogen receptor; GSEA: gene set enrichment analysis; HER2+: human epidermal growth factor receptor 2-positive; IHC: immunohistochemistry; MBC: metastatic breast cancer; MSigDB: Molecular Signatures Database; PARP: poly (ADP-ribose) polymerase.

## Competing interests

RPM, BSW, SR, and DCS are listed as inventors on a patent application to use the BD-L metagene signature.

## Authors' information

Ryan P McMullin Ben S Wittner and Chuanwei Yang are first co-authors. Sridhar Ramaswamy and Dennis C Sgroi are senior co-authors.

## Supplementary Material

Additional file 1 **Assorted Tables.** 1. Cohort characteristics. 2. Probe mapping by figure. 3. GSEA output. 4. Cell line panel *BD-L* values. 5. IPA networks. 6. IPA bio functions. Click here for file

Additional file 2 ***BD-L *value distribution in the discovery cohort.** Wilcoxon tests of the *BD-L* metagene (left panel) and BRCA1 probe sets (middle, right panels) for the primary tumors (primary) and brain metastases (mets) of the HER2+ discovery cohort. Click here for file

Additional file 3 **Correlation of BRCA1 expression with *BD-L *values for the discovery cohort.** Pearson correlation of *BD-L* metagene values and BRCA1 probe set values for all specimens of the HER2+ discovery cohort. Click here for file

Additional file 4 **Distribution of select probe sets across the discovery cohort.** Histograms representing the distribution for the highest differentially expressed probe across all samples in the HER2+ discovery cohort. Each plot highlights a specific gene probe set indicated above the histogram, with each patient’s corresponding expression value highlighted as a red line. Click here for file

Additional file 5 **Linear regression of *BD-L *by cell line viability under increasing concentrations of olaparib.** Linear regression analyses of *BD-L* metagene value by percentage cell line viability following administration of 100 uM temozolomide (TMZ) and indicated concentrations of olaparib. *BD-L* metagene values for the respective cell lines are calculated using a gene expression data set derived from Neve *et al*. [21] (Table 1). Click here for file

Additional file 6 **Linear regression of published BRCA1/2-associated signatures by cell line viability.** Linear regression analyses of BRCA-related signatures by percentage cell line viability following single and combination treatment with 40 nM olaparib and 100 uM temozolomide. **(A,B)** BRCA1-related ovarian cancer signature as described in Konstantinopoulous *et al*. [51] calculated using data sets from (A) Neve *et al*. [21] and (B) Garnett *et al*. [50]; **(C,D)** BRCA1-related breast cancer signature as described in Kote-Jarai *et al*. [53] calculated using data sets from (C) Neve *et al*. and (D) Garnett *et al*.; **(E,F)** BRCA1-related ovarian cancer signature as described in Kote-Jarai *et al*. [52] calculated using data sets from (E) Neve *et al*. and (F) Garnett *et al*.; **(G,H)** BRCA2-related ovarian cancer signature as described in Kote-Jarai *et al.*[52] calculated using data sets from (E) Neve *et al*. and (F) Garnett *et al*. Click here for file

## References

[B1] Leyland-Jones B Human epidermal growth factor receptor 2-positive breast cancer and central nervous system metastases J Clin Oncol 2009 27 5278 5286 10.1200/JCO.2008.19.8481 19770385

[B2] Pienkowski T Zielinski CC Trastuzumab treatment in patients with breast cancer and metastatic CNS disease Ann Oncol 2010 21 917 924 10.1093/annonc/mdp353 19717536

[B3] Weil RJ Palmieri DC Bronder JL Stark AM Steeg PS Breast cancer metastasis to the central nervous system Am J Pathol 2005 167 913 920 10.1016/S0002-9440(10)61180-7 16192626 PMC1603675

[B4] Gabos Z Sinha R Hanson J Chauhan N Hugh J Mackey JR Abdulkarim B Prognostic significance of human epidermal growth factor receptor positivity for the development of brain metastasis after newly diagnosed breast cancer J Clin Oncol 2006 24 5658 5663 10.1200/JCO.2006.07.0250 17102066

[B5] Lin NU Vanderplas A Hughes ME Theriault RL Edge SB Wong YN Blayney DW Niland JC Winer EP Weeks JC Clinicopathologic features, patterns of recurrence, and survival among women with triple-negative breast cancer in the National Comprehensive Cancer Network Cancer 2012 118 5463 5472 10.1002/cncr.27581 22544643 PMC3611659

[B6] Atchley DP Albarracin CT Lopez A Valero V Amos CI Gonzalez-Angulo AM Hortobagyi GN Arun BK Clinical and pathologic characteristics of patients with BRCA-positive and BRCA-negative breast cancer J Clin Oncol 2008 26 4282 4288 10.1200/JCO.2008.16.6231 18779615 PMC6366335

[B7] Gonzalez-Angulo AM Timms KM Liu S Chen H Litton JK Potter J Lanchbury JS Stemke-Hale K Hennessy BT Arun BK Hortobagyi GN Do KA Mills GB Meric-Bernstam F Incidence and outcome of BRCA mutations in unselected patients with triple receptor-negative breast cancer Clin Cancer Res 2011 17 1082 1089 10.1158/1078-0432.CCR-10-2560 21233401 PMC3048924

[B8] Lee LJ Alexander B Schnitt SJ Comander A Gallagher B Garber JE Tung N Clinical outcome of triple negative breast cancer in BRCA1 mutation carriers and noncarriers Cancer 2011 117 3093 3100 10.1002/cncr.25911 21264845 PMC4086795

[B9] Olson EM Abdel-Rasoul M Maly J Wu CS Lin NU Shapiro CL Incidence and risk of central nervous system metastases as site of first recurrence in patients with HER2-positive breast cancer treated with adjuvant trastuzumab Ann Oncol 2013 24 1526 1533 10.1093/annonc/mdt036 23463626 PMC3660080

[B10] Ma XJ Wang Z Ryan PD Isakoff SJ Barmettler A Fuller A Muir B Mohapatra G Salunga R Tuggle JT Tran Y Tran D Tassin A Amon P Wang W Wang W Enright E Stecker K Estepa-Sabal E Smith B Younger J Balis U Michaelson J Bhan A Habin K Baer TM Brugge J Haber DA Erlander MG Sgroi DC A two-gene expression ratio predicts clinical outcome in breast cancer patients treated with tamoxifen Cancer Cell 2004 5 607 616 10.1016/j.ccr.2004.05.015 15193263

[B11] Ma XJ Dahiya S Richardson E Erlander M Sgroi DC Gene expression profiling of the tumor microenvironment during breast cancer progression Breast Cancer Res 2009 11 R7 10.1186/bcr2222 19187537 PMC2687710

[B12] Edgar R Domrachev M Lash AE Gene Expression Omnibus: NCBI gene expression and hybridization array data repository Nucleic Acids Res 2002 30 207 210 10.1093/nar/30.1.207 11752295 PMC99122

[B13] Gene Expression Omnibus [http://www.ncbi.nlm.nih.gov/geo/query/acc.cgi?acc=GSE43837]

[B14] Bioconductor [http://www.bioconductor.org]

[B15] Gene Set Enrichment Analysis [http://www.broadinstitute.org/gsea]

[B16] Holm S A simple sequentially rejective multiple test procedure Scandinavian J Stat 1979 6 65 70

[B17] The Broad Institute of MIT and Harvard [http://www.broadinstitute.org]

[B18] NKI Cohort Contig Annotation [http://archive-nl.com/page/2361770/2013-06-27/http://bioinformatics.nki.nl/data/van-t-Veer_Nature_2002/?C=S;O=A]

[B19] Akbari M Donenberg T Lunn J Curling D Turnquest T Krill-Jackson E Zhang S Narod S Hurley J The spectrum of BRCA1 and BRCA2 mutations in breast cancer patients in the Bahamas Clin Genet 2014 85 64 67 10.1111/cge.12132 23458327

[B20] Rozen S Skaletsky H Primer3 on the WWW for general users and for biologist programmers Methods Mol Biol 2000 132 365 386 10547847 10.1385/1-59259-192-2:365

[B21] Neve RM Chin K Fridlyand J Yeh J Baehner FL Fevr T Clark L Bayani N Coppe JP Tong F Speed T Spellman PT DeVries S Lapuk A Wang NJ Kuo WL Stilwell JL Pinkel D Albertson DG Waldman FM McCormick F Dickson RB Johnson MD Lippman M Ethier S Gazdar A Gray JW A collection of breast cancer cell lines for the study of functionally distinct cancer subtypes Cancer Cell 2006 10 515 527 10.1016/j.ccr.2006.10.008 17157791 PMC2730521

[B22] Subramanian A Tamayo P Mootha VK Mukherjee S Ebert BL Gillette MA Paulovich A Pomeroy SL Golub TR Lander ES Mesirov JP Gene set enrichment analysis: a knowledge-based approach for interpreting genome-wide expression profiles Proc Natl Acad Sci U S A 2005 102 15545 15550 10.1073/pnas.0506580102 16199517 PMC1239896

[B23] Welcsh PL Lee MK Gonzalez-Hernandez RM Black DJ Mahadevappa M Swisher EM Warrington JA King MC BRCA1 transcriptionally regulates genes involved in breast tumorigenesis Proc Natl Acad Sci U S A 2002 99 7560 7565 10.1073/pnas.062181799 12032322 PMC124283

[B24] Armes JE Trute L White D Southey MC Hammet F Tesoriero A Hutchins AM Dite GS McCredie MR Giles GG Hopper JL Venter DJ Distinct molecular pathogeneses of early-onset breast cancers in BRCA1 and BRCA2 mutation carriers: a population-based study Cancer Res 2011–2017 1999 59 10213514

[B25] Robson M Rajan P Rosen PP Gilewski T Hirschaut Y Pressman P Haas B Norton L Offit K BRCA-associated breast cancer: absence of a characteristic immunophenotype Cancer Res 1839–1842 1998 58 9581822

[B26] Vaziri SA Krumroy LM Elson P Budd GT Darlington G Myles J Tubbs RR Casey G Breast tumor immunophenotype of BRCA1-mutation carriers is influenced by age at diagnosis Clin Cancer Res 1937–1945 2001 7 11448907

[B27] Plon SE Eccles DM Easton D Foulkes WD Genuardi M Greenblatt MS Hogervorst FB Hoogerbrugge N Spurdle AB Tavtigian SV Sequence variant classification and reporting: recommendations for improving the interpretation of cancer susceptibility genetic test results Hum Mutat 2008 29 1282 1291 10.1002/humu.20880 18951446 PMC3075918

[B28] Al-Mulla F Abdulrahman M Varadharaj G Akhter N Anim JT BRCA1 gene expression in breast cancer: a correlative study between real-time RT-PCR and immunohistochemistry J Histochem Cytochem 2005 53 621 629 10.1369/jhc.4A6544.2005 15872055

[B29] Zhang XH Wang Q Gerald W Hudis CA Norton L Smid M Foekens JA Massague J Latent bone metastasis in breast cancer tied to Src-dependent survival signals Cancer Cell 2009 16 67 78 10.1016/j.ccr.2009.05.017 19573813 PMC2749247

[B30] Van't Veer LJ Dai H van de Vijver MJ He YD Hart AA Mao M Peterse HL van der Kooy K Marton MJ Witteveen AT Schreiber GJ Kerkhoven RM Roberts C Linsley PS Bernards R Friend SH Gene expression profiling predicts clinical outcome of breast cancer Nature 2002 415 530 536 10.1038/415530a 11823860

[B31] Albiges L Andre F Balleyguier C Gomez-Abuin G Chompret A Delaloge S Spectrum of breast cancer metastasis in BRCA1 mutation carriers: highly increased incidence of brain metastases Ann Oncol 1846–1847 2005 16 10.1093/annonc/mdi351 15972278

[B32] van de Vijver MJ He YD van't Veer LJ Dai H Hart AA Voskuil DW Schreiber GJ Peterse JL Roberts C Marton MJ Parrish M Atsma D Witteveen A Glas A Delahaye L van der Velde T Bartelink H Rodenhuis S Rutgers ET Friend SH Bernards R A gene-expression signature as a predictor of survival in breast cancer N Engl J Med 1999–2009 2002 347 10.1056/NEJMoa021967 12490681

[B33] Wang Y Klijn JG Zhang Y Sieuwerts AM Look MP Yang F Talantov D Timmermans M Meijer-van Gelder ME Yu J Jatkoe T Berns EM Atkins D Foekens JA Gene-expression profiles to predict distant metastasis of lymph-node-negative primary breast cancer Lancet 2005 365 671 679 10.1016/S0140-6736(05)17947-1 15721472

[B34] Minn AJ Gupta GP Siegel PM Bos PD Shu W Giri DD Viale A Olshen AB Gerald WL Massague J Genes that mediate breast cancer metastasis to lung Nature 2005 436 518 524 10.1038/nature03799 16049480 PMC1283098

[B35] Bos PD Zhang XH Nadal C Shu W Gomis RR Nguyen DX Minn AJ van de Vijver MJ Gerald WL Foekens JA Massague J Genes that mediate breast cancer metastasis to the brain Nature 2009 459 1005 1009 10.1038/nature08021 19421193 PMC2698953

[B36] Clark GM Sledge GW Jr Osborne CK McGuire WL Survival from first recurrence: relative importance of prognostic factors in 1,015 breast cancer patients J Clin Oncol 1987 5 55 61 3806159 10.1200/JCO.1987.5.1.55

[B37] Audeh MW Carmichael J Penson RT Friedlander M Powell B Bell-McGuinn KM Scott C Weitzel JN Oaknin A Loman N Lu K Schmutzler RK Matulonis U Wickens M Tutt A Oral poly(ADP-ribose) polymerase inhibitor olaparib in patients with BRCA1 or BRCA2 mutations and recurrent ovarian cancer: a proof-of-concept trial Lancet 2010 376 245 251 10.1016/S0140-6736(10)60893-8 20609468

[B38] Tutt A Robson M Garber JE Domchek SM Audeh MW Weitzel JN Friedlander M Arun B Loman N Schmutzler RK Wardley A Mitchell G Earl H Wickens M Carmichael J Oral poly(ADP-ribose) polymerase inhibitor olaparib in patients with BRCA1 or BRCA2 mutations and advanced breast cancer: a proof-of-concept trial Lancet 2010 376 235 244 10.1016/S0140-6736(10)60892-6 20609467

[B39] Plummer R Poly(ADP-ribose) polymerase inhibition: a new direction for BRCA and triple-negative breast cancer? Breast Cancer Res 2011 13 218 10.1186/bcr2877 21884642 PMC3236327

[B40] Rouleau M Patel A Hendzel MJ Kaufmann SH Poirier GG PARP inhibition: PARP1 and beyond Nat Rev Cancer 2010 10 293 301 10.1038/nrc2812 20200537 PMC2910902

[B41] Lord CJ McDonald S Swift S Turner NC Ashworth A A high-throughput RNA interference screen for DNA repair determinants of PARP inhibitor sensitivity DNA Repair (Amst) 2008 7 2010 2019 10.1016/j.dnarep.2008.08.014 18832051

[B42] McCabe N Turner NC Lord CJ Kluzek K Bialkowska A Swift S Giavara S O'Connor MJ Tutt AN Zdzienicka MZ Smith GC Ashworth A Deficiency in the repair of DNA damage by homologous recombination and sensitivity to poly(ADP-ribose) polymerase inhibition Cancer Res 2006 66 8109 8115 10.1158/0008-5472.CAN-06-0140 16912188

[B43] Turner NC Lord CJ Iorns E Brough R Swift S Elliott R Rayter S Tutt AN Ashworth A A synthetic lethal siRNA screen identifying genes mediating sensitivity to a PARP inhibitor Embo J 2008 27 1368 1377 10.1038/emboj.2008.61 18388863 PMC2374839

[B44] Forster MD Dedes KJ Sandhu S Frentzas S Kristeleit R Ashworth A Poole CJ Weigelt B Kaye SB Molife LR Treatment with olaparib in a patient with PTEN-deficient endometrioid endometrial cancer Nat Rev Clin Oncol 2011 8 302 306 10.1038/nrclinonc.2011.42 21468130

[B45] Donawho CK Luo Y Luo Y Penning TD Bauch JL Bouska JJ Bontcheva-Diaz VD Cox BF DeWeese TL Dillehay LE Ferguson DC Ghoreishi-Haack NS Grimm DR Guan R Han EK Holley-Shanks RR Hristov B Idler KB Jarvis K Johnson EF Kleinberg LR Klinghofer V Lasko LM Liu X Marsh KC McGonigal TP Meulbroek JA Olson AM Palma JP Rodriguez LE ABT-888, an orally active poly(ADP-ribose) polymerase inhibitor that potentiates DNA-damaging agents in preclinical tumor models Clin Cancer Res 2007 13 2728 2737 10.1158/1078-0432.CCR-06-3039 17473206

[B46] Liu X Shi Y Guan R Donawho C Luo Y Palma J Zhu GD Johnson EF Rodriguez LE Ghoreishi-Haack N Jarvis K Hradil VP Colon-Lopez M Cox BF Klinghofer V Penning T Rosenberg SH Frost D Giranda VL Luo Y Potentiation of temozolomide cytotoxicity by poly(ADP)ribose polymerase inhibitor ABT-888 requires a conversion of single-stranded DNA damages to double-stranded DNA breaks Mol Cancer Res 2008 6 1621 1629 18922977 10.1158/1541-7786.MCR-08-0240

[B47] Miknyoczki SJ Jones-Bolin S Pritchard S Hunter K Zhao H Wan W Ator M Bihovsky R Hudkins R Chatterjee S Klein-Szanto A Dionne C Ruggeri B Chemopotentiation of temozolomide, irinotecan, and cisplatin activity by CEP-6800, a poly(ADP-ribose) polymerase inhibitor Mol Cancer Ther 2003 2 371 382 10.4161/cbt.2.4.460 12700281

[B48] Plummer R Jones C Middleton M Wilson R Evans J Olsen A Curtin N Boddy A McHugh P Newell D Harris A Johnson P Steinfeldt H Dewji R Wang D Robson L Calvert H Phase I study of the poly(ADP-ribose) polymerase inhibitor, AG014699, in combination with temozolomide in patients with advanced solid tumors Clin Cancer Res 2008 14 7917 7923 10.1158/1078-0432.CCR-08-1223 19047122 PMC2652879

[B49] Fu D Calvo JA Samson LD Balancing repair and tolerance of DNA damage caused by alkylating agents Nat Rev Cancer 2012 12 104 120 22237395 10.1038/nrc3185 PMC3586545

[B50] Garnett MJ Edelman EJ Heidorn SJ Greenman CD Dastur A Lau KW Greninger P Thompson IR Luo X Soares J Liu Q Iorio F Surdez D Chen L Milano RJ Bignell GR Tam AT Davies H Stevenson JA Barthorpe S Lutz SR Kogera F Lawrence K McLaren-Douglas A Mitropoulos X Mironenko T Thi H Richardson L Zhou W Jewitt F Systematic identification of genomic markers of drug sensitivity in cancer cells Nature 2012 483 570 575 10.1038/nature11005 22460902 PMC3349233

[B51] Konstantinopoulos PA Spentzos D Karlan BY Taniguchi T Fountzilas E Francoeur N Levine DA Cannistra SA Gene expression profile of BRCAness that correlates with responsiveness to chemotherapy and with outcome in patients with epithelial ovarian cancer J Clin Oncol 2010 28 3555 3561 10.1200/JCO.2009.27.5719 20547991 PMC2917311

[B52] Kote-Jarai Z Matthews L Osorio A Shanley S Giddings I Moreews F Locke I Evans DG Eccles D Williams RD Girolami M Campbell C Eeles R Accurate prediction of BRCA1 and BRCA2 heterozygous genotype using expression profiling after induced DNA damage Clin Cancer Res 2006 12 3896 3901 10.1158/1078-0432.CCR-05-2805 16818684

[B53] Kote-Jarai Z Williams RD Cattini N Copeland M Giddings I Wooster R tePoele RH Workman P Gusterson B Peacock J Gui G Campbell C Eeles R Gene expression profiling after radiation-induced DNA damage is strongly predictive of BRCA1 mutation carrier status Clin Cancer Res 2004 10 958 963 10.1158/1078-0432.CCR-1067-3 14871973

[B54] Gelmon KA Tischkowitz M Mackay H Swenerton K Robidoux A Tonkin K Hirte H Huntsman D Clemons M Gilks B Yerushalmi R Macpherson E Carmichael J Oza A Olaparib in patients with recurrent high-grade serous or poorly differentiated ovarian carcinoma or triple-negative breast cancer: a phase 2, multicentre, open-label, non-randomised study Lancet Oncol 2011 12 852 861 10.1016/S1470-2045(11)70214-5 21862407

