# Supplementary figures and images for: A BRCA1 deficient-like signature is enriched in breast cancer brain metastases and predicts DNA damage-induced poly (ADP-ribose) polymerase inhibitor sensitivity
Source: Breast Cancer Res. 2014 Mar 14;16(2):R25. doi: 10.1186/bcr3625 (PMC4053087; doi:10.1186/bcr3625)

Wilcoxon p=0.0082

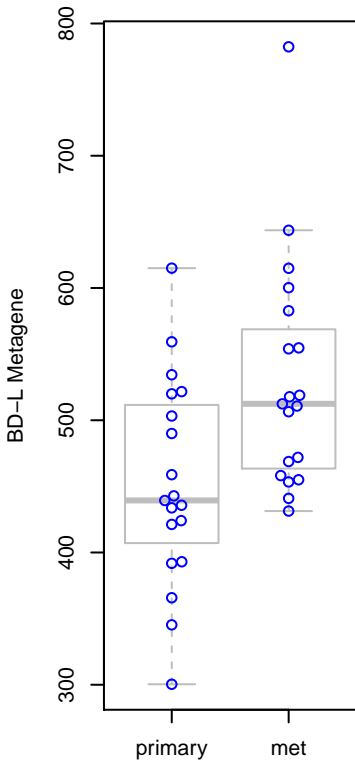

Wilcoxon p=0.89

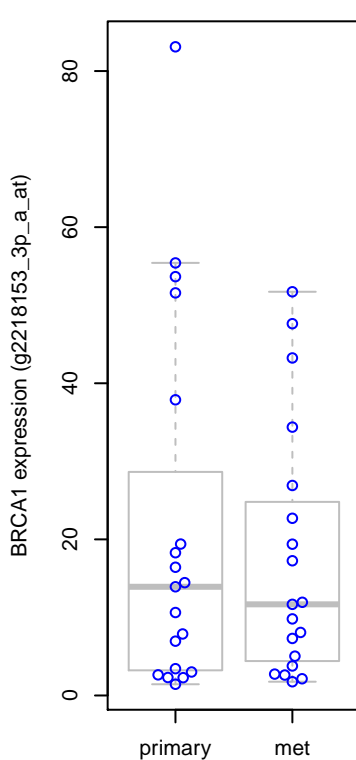

Wilcoxon p=0.21

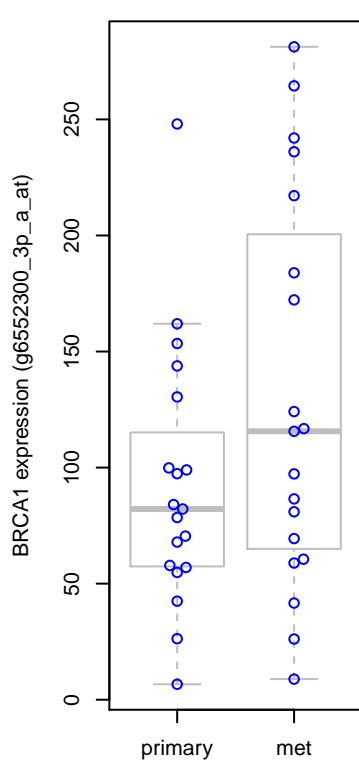

Supplement: Additional file 2 — BD-L value distribution in the discovery cohort. Wilcoxon tests of the BD-L metagene (left panel) and BRCA1 probe sets (middle, right panels) for the primary tumors (primary) and brain metastases (mets) of the HER2+ discovery cohort. [file bcr3625-S2.pdf]

**Pearson p-value = 0.06  $r = -0.31$**

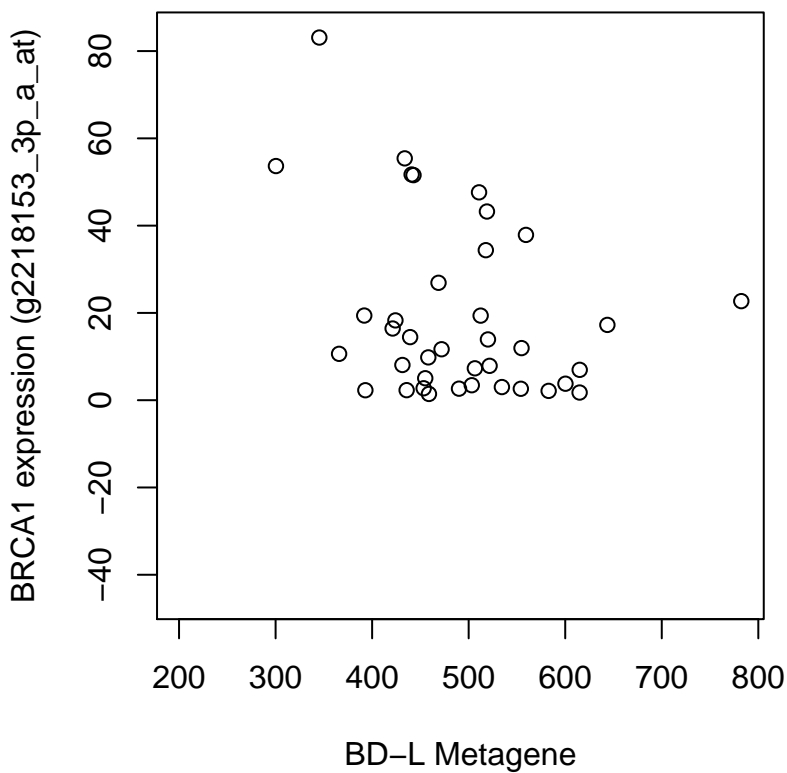

**Pearson p-value = 0.14  $r = 0.24$**

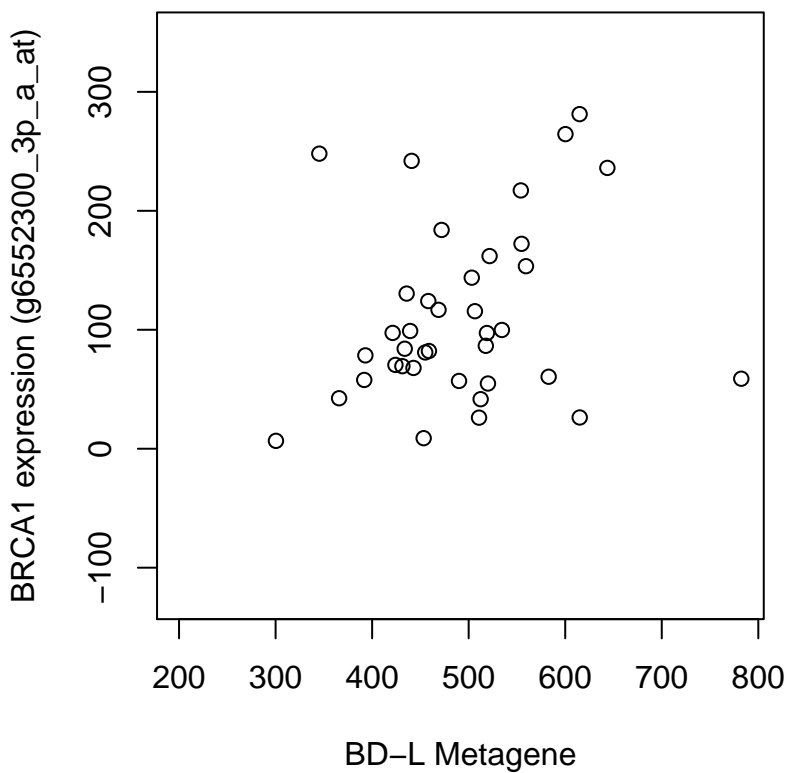

Supplement: Additional file 3 — Correlation of BRCA1 expression with BD-L values for the discovery cohort. Pearson correlation of BD-L metagene values and BRCA1 probe set values for all specimens of the HER2+ discovery cohort. [file bcr3625-S3.pdf]

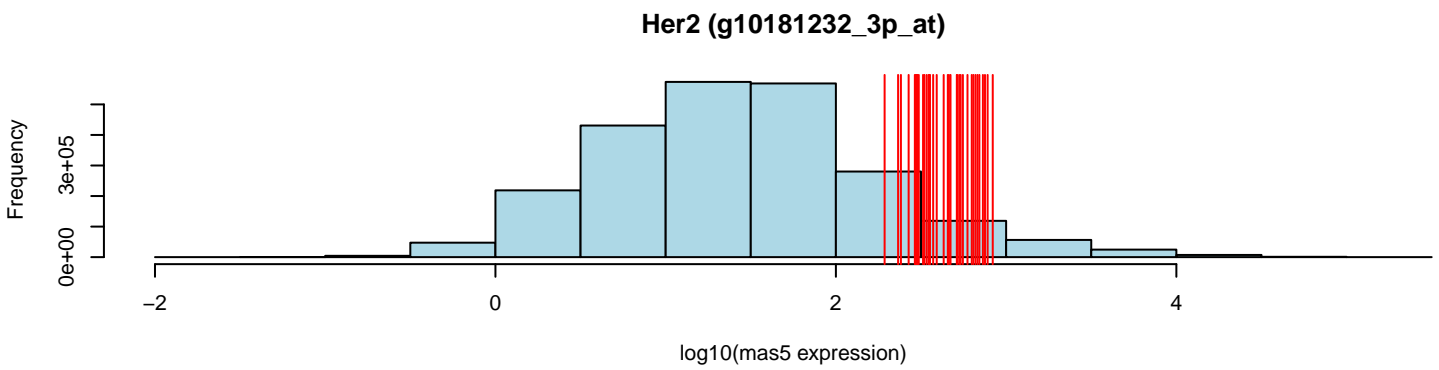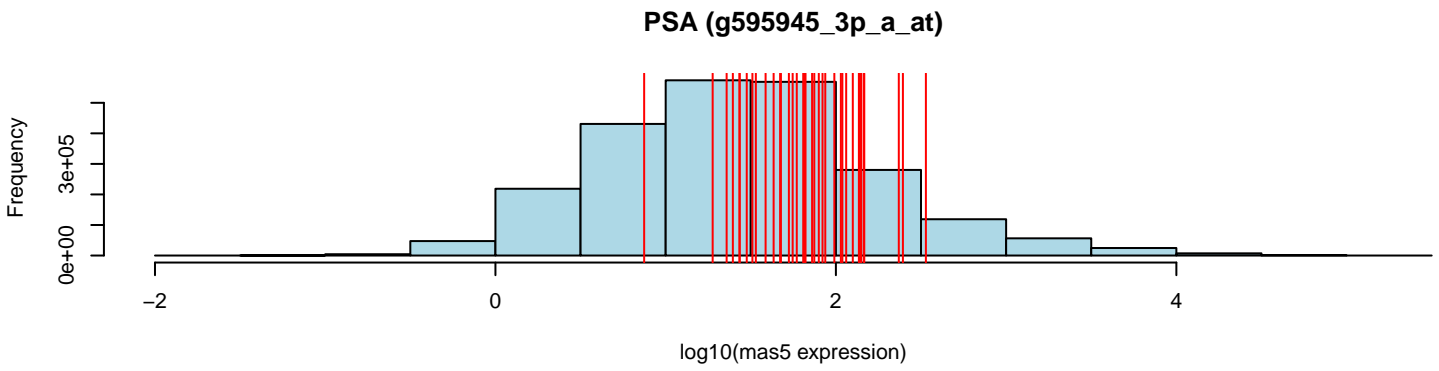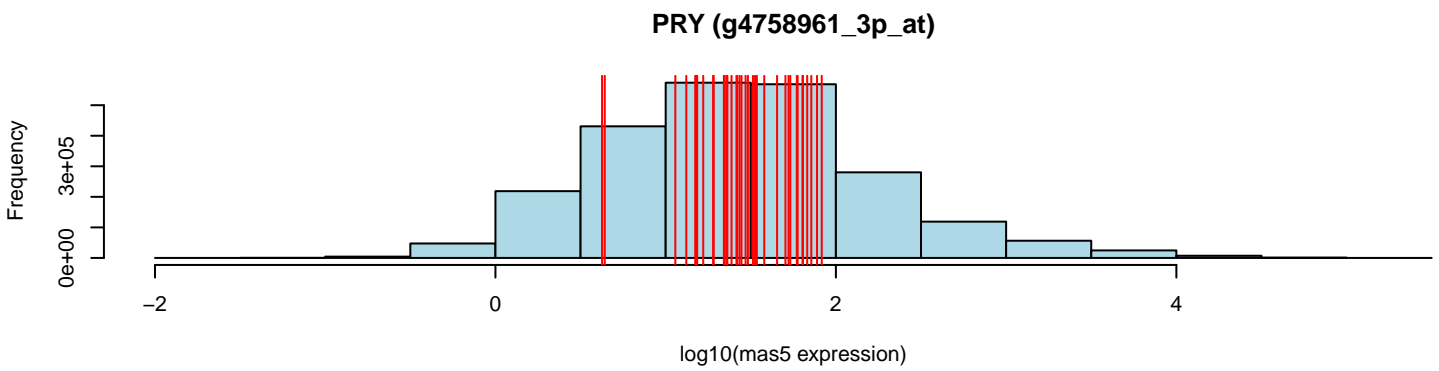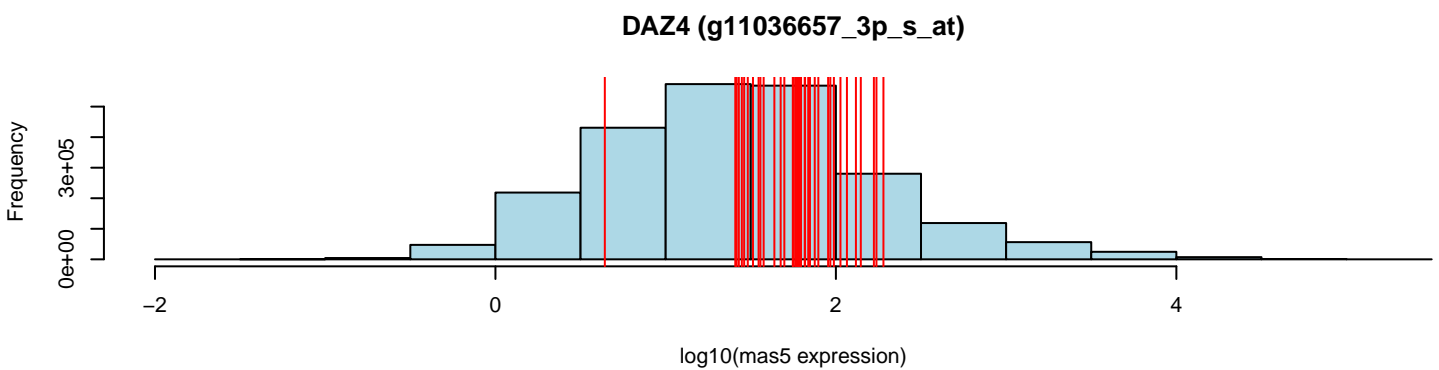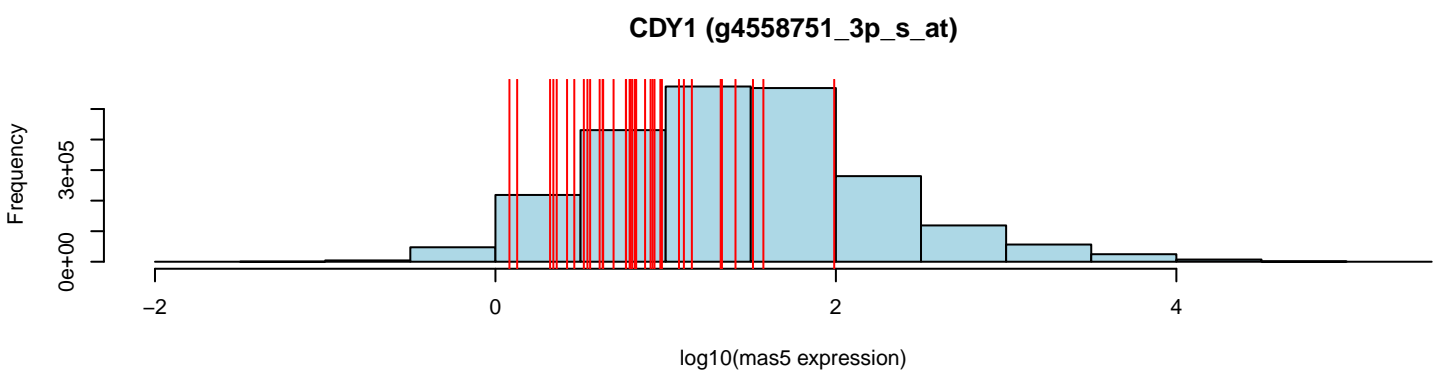

Supplement: Additional file 4 — Distribution of select probe sets across the discovery cohort. Histograms representing the distribution for the highest differentially expressed probe across all samples in the HER2+ discovery cohort. Each plot highlights a specific gene probe set indicated above the histogram, with each patient’s corresponding expression value highlighted as a red line. [file bcr3625-S4.pdf]

**TMZ + 5 nM Olaparib**

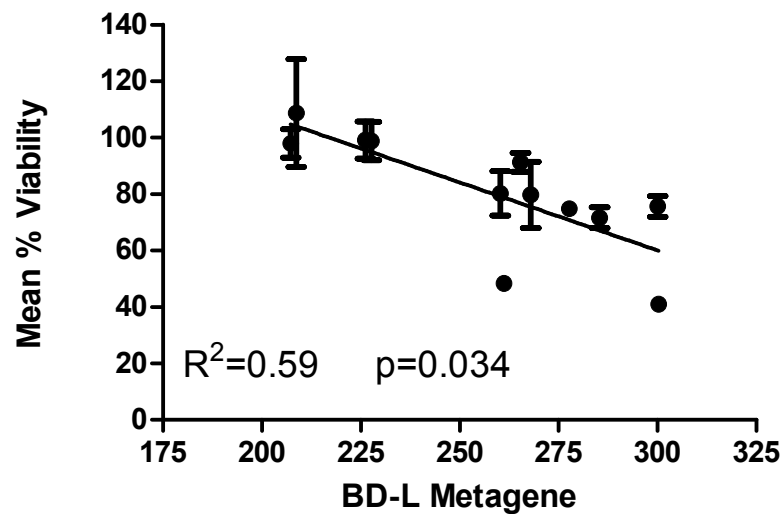

**TMZ + 10 nM Olaparib**

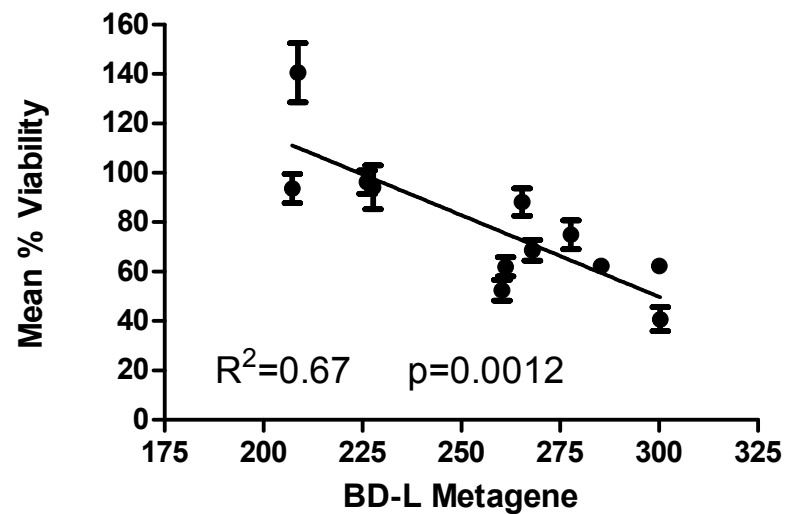

**TMZ + 20 nM Olaparib**

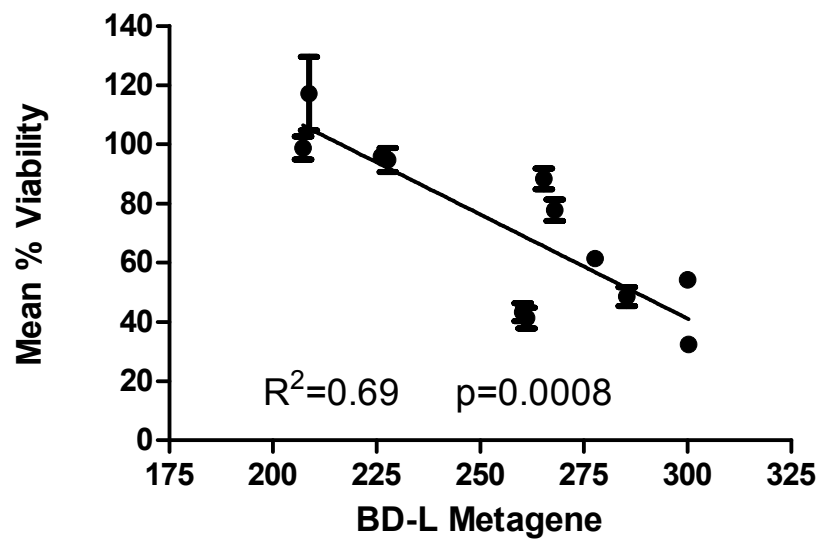

Supplement: Additional file 5 — Linear regression of BD-L by cell line viability under increasing concentrations of olaparib. Linear regression analyses of BD-L metagene value by percentage cell line viability following administration of 100 uM temozolomide (TMZ) and indicated concentrations of olaparib. BD-L metagene values for the respective cell lines are calculated using a gene expression data set derived from Neve et al. [21] (Table 1). [file bcr3625-S5.pdf]

## Slide 1
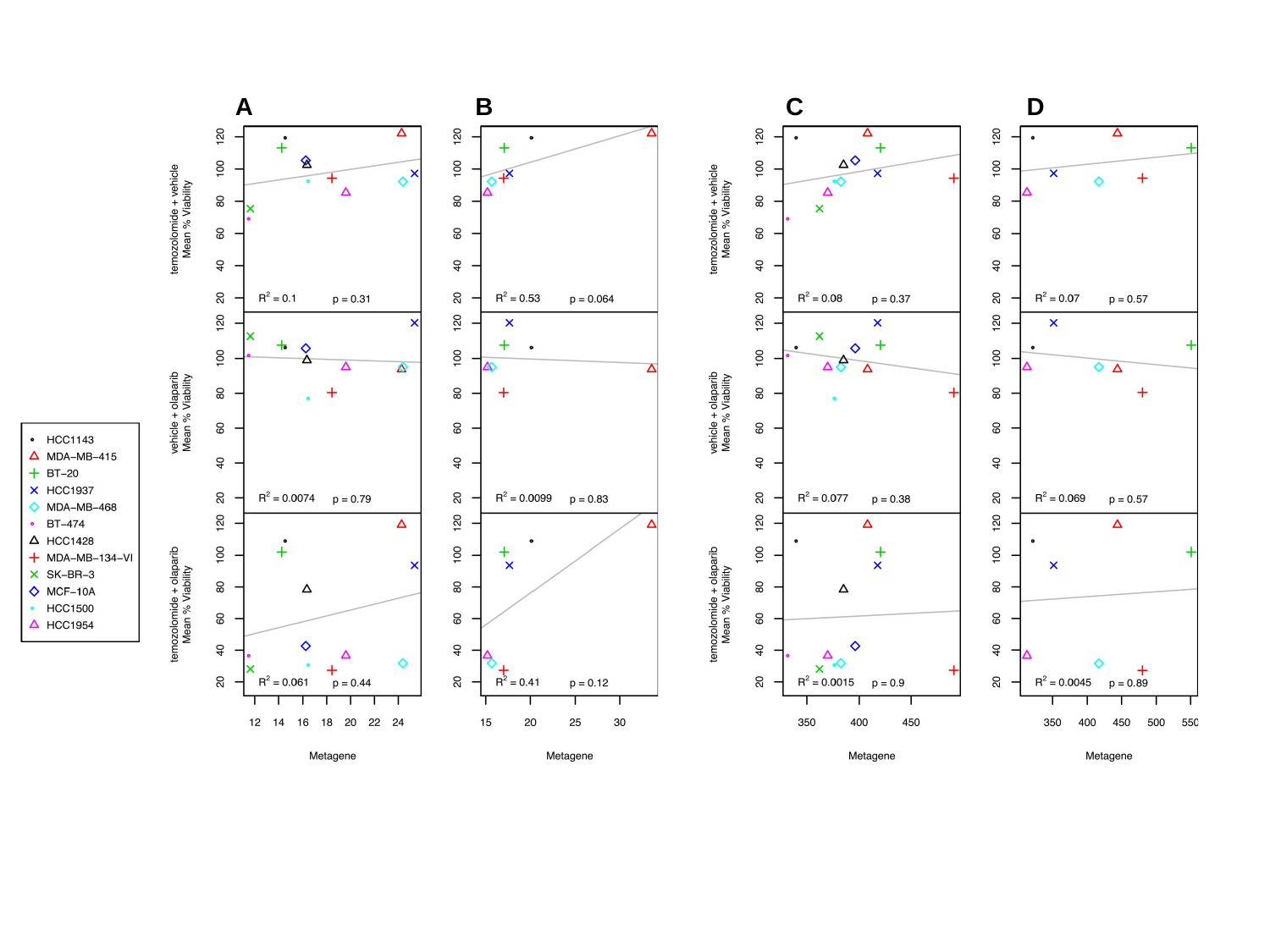

A B C D

## Slide 2
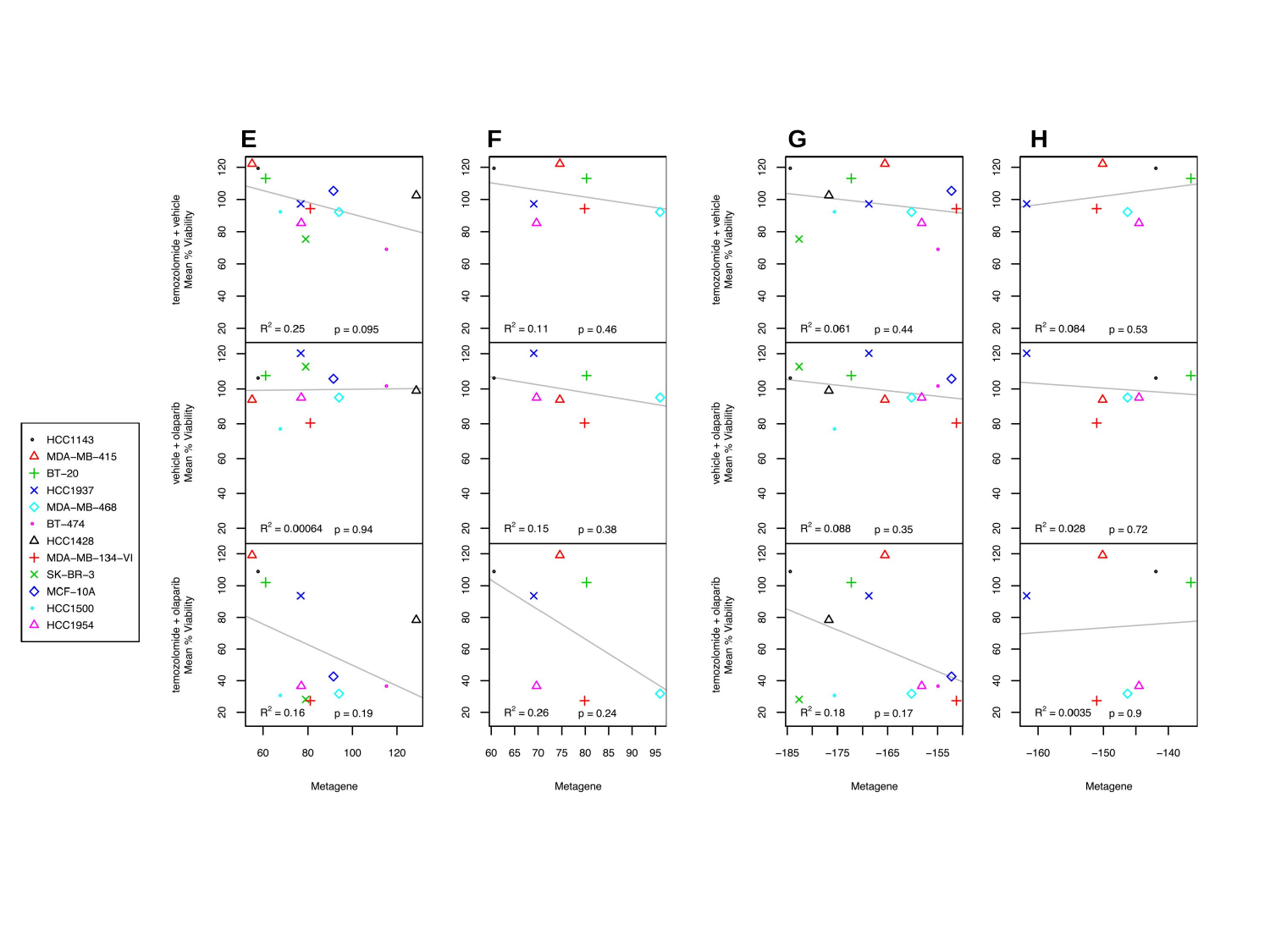

E F G H

Supplement: Additional file 6 — Linear regression of published BRCA1/2-associated signatures by cell line viability. Linear regression analyses of BRCA-related signatures by percentage cell line viability following single and combination treatment with 40 nM olaparib and 100 uM temozolomide. (A,B) BRCA1-related ovarian cancer signature as described in Konstantinopoulous et al. [51] calculated using data sets from (A) Neve et al. [21] and (B) Garnett et al. [50]; (C,D) BRCA1-related breast cancer signature as described in Kote-Jarai et al. [53] calculated using data sets from (C) Neve et al. and (D) Garnett et al.; (E,F) BRCA1-related ovarian cancer signature as described in Kote-Jarai et al. [52] calculated using data sets from (E) Neve et al. and (F) Garnett et al.; (G,H) BRCA2-related ovarian cancer signature as described in Kote-Jarai et al.[52] calculated using data sets from (E) Neve et al. and (F) Garnett et al. [file bcr3625-S6.ppt]
